# Let her shine: Empowering women and closing India’s gender gap in STEM

**DOI:** 10.1371/journal.pmed.1004937

**Published:** 2026-02-10

**Authors:** Aparajita Chattopadhyay

**Affiliations:** Department of Population and Development, International Institute for Population Sciences, Mumbai, India

## Abstract

In recognition of International day of women and girls in STEM, this Editorial by Aparajita Chattopadhyay reflects on the gender gap in STEM in India, outlining socio-cultural and infrastructural factors, recent promising trends, and what is needed to continue these transformations.

## Teaser

Despite globally increasing numbers of women in the STEM workforce, levels still remain suboptimal. Closing the gender gap in an emerging economic power such as India, requires a cultural shift, active support of girls’ education, and more secure job opportunities for graduates.

The International Day of Women and Girls in Science, Technology, Engineering, and Mathematics (STEM) not only celebrates the achievements and contributions of women in these fields but also underscores the urgent need to recognize and address the persistent gender gap that remains today. Despite increasing participation—with women accounting for ~28% of the global STEM workforce in 2024 compared to 21% in 2016 [1]—there is still progress to be made. We should be empowering women to pursue a career in STEM by encouraging girls’ participation in STEM education from an early age and removing barriers that limit women’s career progression and leadership in science. Women’s contributions (which have historically often been overlooked) should also be celebrated, and creating inclusive scientific and research ecosystems should be a priority.

No country in the world is fully gender-equal, particularly in the field of STEM, and India is no exception. I wish to speak from the context of India where my heart lies and whose society I know well. The success of a girl in STEM requires open-minded parenting, gender-neutral school curricula, progressive and gender-sensitive social environments, and systemic mechanisms that allow women and girls to raise their voices without fear. Investing in girls’ education and modern skills, particularly in STEM, is not an act of charity, but a productive investment in society [2].

Let us look at some statistics that are both encouraging and concerning. The proportion of women enrolling in STEM courses in India has risen from 38% in 2014–2015 to about 43% in 2021–2022 [3], placing India well ahead of many developed nations globally. Despite some recent encouraging trends, women’s workforce participation remains suboptimal. Though women constitute more than 40% of STEM graduates in India, only about 20%–30% of professionals in the STEM workforce are women, as well as globally [1,2,4,5]. Less than 20% of women are scientists and faculty staff in research institutions and higher education in India [6]. While Indian women demonstrate remarkable capabilities in STEM, systemic challenges including workplace hazards and gaps in infrastructure, often restrict their professional opportunities and career progression. For example, in medical education, women represent ~51% of MBBS students, yet female graduates show higher attrition rates and account for only 29% of allopathic doctors [7]. Factors such as violence against healthcare workers, excessive occupational stress among young doctors and care providers, and unsafe working environments contribute to this attrition [8]. In scientific research, long periods of uncertain career prospects often force women to choose between pursuing research, entering the job market, or getting married, which can make their professional growth undefined. Women often have to endure casual mockery and dismissive remarks that question their right to study, work and succeed.

The persistence of gender gaps in India is driven in part by deep-rooted socio-cultural factors, although recent years have seen an increased emphasis on incentivising female participation in higher education. Early marriage in India, though declining steadily, continues to be a concern, with nearly one in four women (23%) aged 20–24 marrying before the age of 18 [9]. These societal pressures force women out of education, while boys’ education is prioritized. Further, dowry is so deeply entrenched in Indian society that its demand and fulfillment are normalized as symbols of prestige, allowing such offences to persist beyond legal accountability. The persistent juggle between individualistic aspirations and altruistic expectations has long left Indian women navigating a complex and often conflicting social terrain. For decades or even centuries, many people have internalized a functionalist stratification of society—one that was built on women’s sacrifice, silence, and unpaid labor. Interestingly, women in ancient India contributed to mathematics, astronomy, medicine, and philosophy, often through family and intellectual debates, yet their work was rarely documented, but oral traditions and mathematical treatises show that women actively participated in knowledge creation. The current challenge, therefore, lies in translating intent into sustained action—through policies, institutional reform, and everyday practices that collectively enable girls not merely to partake, but to lead.

Although India’s public spending on education is appreciable (around 4.1% of GDP), investment in research and development remains below 1% of GDP, constraining knowledge creation, applied research, and industry–academia collaboration. Without sufficient funding, India’s education and research system may be unable to demonstrate its full capabilities. India therefore urgently requires substantial investment in innovation, technological advancement across industries, and sustained expansion of STEM job markets. Yet several structural contradictions persist. For example, faculty and research positions in public universities are often vacant for years; according to the 2025−2026 *Demands for Grants of the Department of Higher Education* report [10], the vacancy rate for professorial positions is a staggering 56%. Moreover, a large proportion of graduates from the country’s premier technology institutions end up taking jobs unrelated to their fields of study; recent trends in India reveal that highly educated individuals are increasingly applying for low-paying government jobs. According to the 2024 *India Employment Report* [11], in a state police department, more than 93,000 candidates applying for jobs included 3,700 PhD holders, 5,000 graduates, and 28,000 postgraduates, despite these qualifications not being required. Job prospects are particularly bleak for women: In 2024, 59% of female graduates were neither working nor seeking work, with care/home commitments being the primary reason [12].

However, the Indian landscape is changing, perhaps more rapidly than ever before. India matters to the world in several ways, from supplying a vast pool of skilled human resources to serving as a large and dynamic consumer market. It needs to demonstrate that its education and research system is capable of nurturing the finest minds: minds that innovate, inspire, and generate solutions at scale for billions. The recently launched National Education Policy in India is aimed at promoting high-quality, multidisciplinary, and application-oriented STEM education, with early exposure to experiential learning and emerging technologies [13]. The Government of India runs various science and technology schemes to support women researchers and technologists with a focus on career continuity, innovation, collaboration, and socio-economic empowerment [14]. This is complemented by non-governmental initiatives, such as “Educate Girls,” which is aimed at improving access and quality of education for girls, particularly in rural areas. Alongside scholarships, skilled training, internships and mentorships, women of the modern era are increasingly able to assert autonomy over their life courses. This is reflected in a growing proportion of women remaining unmarried into their late twenties and a rapidly declining fertility rate in India. Gaps in childcare support, the growing nuclearization of families, and the unequal gender distribution of unpaid care, are perhaps also gradually discouraging young women from not choosing early marriage and motherhood. These shifts signal a redefinition of agency, choice, and self-worth in a transforming society.

Women in Indian culture are often referred to as *“Dashabhuja” (10 armed)*—a symbol of strength, resilience, and the ability to perform multiple roles with grace. India takes pride in its women spanning ancient scholars to modern scientists, educators, medical pioneers, and leaders who have consistently demonstrated excellence in various fields despite many constraints, forming a powerful legacy that continues to inspire the nation. When Indian female scientists can send rockets into space, lead transformative movements for girls’ education and social welfare, inspire millions through entrepreneurship and industry, and significantly contribute to the development of COVID-19 vaccines, this demonstrates that this country has the scientific talent and capability to innovate at the highest global level; there should be no second thought about enabling every girl to step forward with confidence and emerge with flying colors.

## Abbreviation:

STEM: Science, Technology, Engineering, and Mathematics
